# Trends in Medical Debt During the COVID-19 Pandemic

**DOI:** 10.1001/jamahealthforum.2022.1031

**Published:** 2022-05-20

**Authors:** Benedict Guttman-Kenney, Raymond Kluender, Neale Mahoney, Francis Wong, Xuyang Xia, Wesley Yin

**Affiliations:** 1 University of Chicago Booth School of Business, Chicago, Illinois; 2 Harvard Business School, Harvard University, Boston, Massachusetts; 3 Department of Economics, Stanford University, Stanford, California; 4 National Bureau of Economic Research, Cambridge, Massachusetts; 5 Department of Economics, Duke University, Durham, North Carolina; 6 Luskin School of Public Affairs, University of California, Los Angeles, Los Angeles

## Abstract

This cross-sectional study evaluates the association between the COVID-19 pandemic and reported new medical debt from 2018 to 2021 across the US.

## Introduction

In the US, with millions of COVID-19 hospitalizations, considerable job losses, and corresponding losses in employer-sponsored health insurance, the COVID-19 pandemic has raised concerns about a substantial increase in medical debt.^1^ However, the decrease in elective medical procedures^2^; passage of legislation that partially shields households from COVID-19–related medical costs^3^; and large expansion of the social safety net,^4^ including increased funding for Medicaid and health insurance exchanges,^5^ may have offset any potential medical debt increase. To better understand these factors, we analyzed trends in medical debt from January 2018 to September 2021 and their associations with local measures of pandemic severity.

## Methods

In this cross-sectional study, we measured medical debt in collections using a nationally representative, randomly selected 10% panel of persons with credit reports maintained by TransUnion, a nationwide credit reporting agency. The University of Chicago Institutional Review Board deemed this analysis of deidentified credit bureau data not to be human participant research. We followed the STROBE reporting guideline.

We supplemented data from January 2018 to June 2020, which were identical to those used in a previous medical debt study,^6^ with newly available data through September 2021. Medical debt is typically reported at least 180 days after the bill was incurred; thus, these data largely reflect medical care provided through March 2021. The 10% panel yielded a sample of 1.457 billion person-month observations and 37 million unique persons.

The main outcome was the mean quarterly flow of medical debt, defined as the amount of new debt listed on credit reports during the preceding 3 months, averaged over persons with credit reports. For comparison, we constructed an analogous measure of the flow of nonmedical debt in collections. The data included zip codes. To analyze trends by income, we assigned each zip code to its population-weighted income quintile using estimates from the 5-year American Community Survey (2015-2019) and then plotted statistics on the quarter 3 flow of medical debt by year.

We examined the sample-weighted association between county-level percentage changes in medical debt from September 2019 to September 2021 and the cumulative COVID-19 infection and vaccination rates through September 2021 using data from the *New York Times*; the change in unemployment rate from September 2019 to the peak of the pandemic (April 2020 for most counties) using data from the US Census Bureau; and the change in consumer spending between January 2020 and September 2021 using data from Opportunity Insights. Details on sample and variable construction appear in the eMethods in the Supplement.

## Results

**Figure 1.**
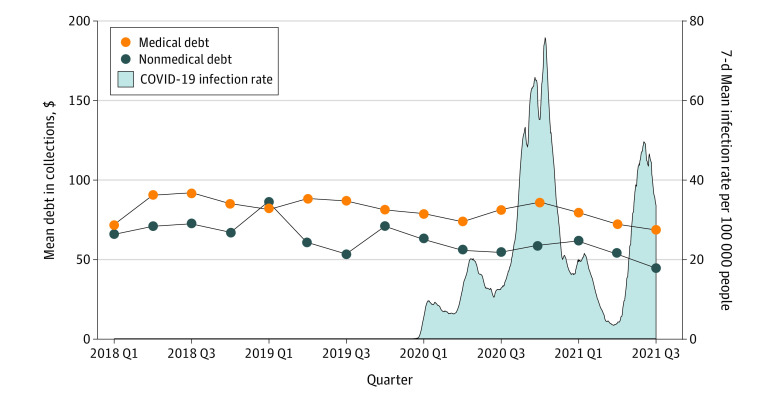
Quarterly Flow of Medical Debt in Collections and COVID-19 Infection Rate Mean quarterly flow of debt was the amount of new debt listed on credit reports in the past 3 months, averaged over all individuals with a credit report. COVID-19 infection rate was the rolling 7-day mean per 100 000 people.

**Figure 2.**
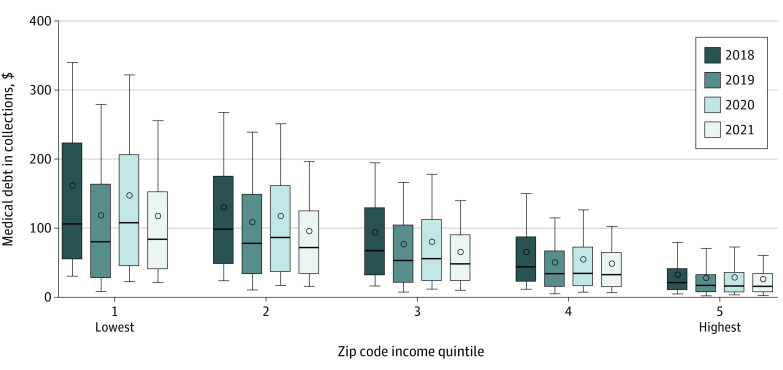
Quarter 3 Flow of Medical Debt by Zip Code Income Quintile Dots indicate means; horizontal lines, medians; boxes, IQRs; whiskers, range (10th-90th percentile) for mean zip code–level quarter 3 debt flow.

Medical and nonmedical debt during the pandemic followed the prepandemic downward trends (Figure 1), with proportionally similar declines across zip code income quintiles (Figure 2). There was no statistically significant association between the percentage change in medical debt and the measures of pandemic severity. The Pearson correlations were small in absolute value: 0.037 with COVID-19 infection rate, −0.054 with COVID-19 vaccination rate, 0.021 with change in unemployment rate, and 0.018 with change in consumer spending.

## Discussion

We found no evidence of a net association between the COVID-19 pandemic and medical debt, overall or across areas with different incomes and pandemic severity. These results are consistent with any increase in medical debt being offset by decreases in elective medical procedures and new health care–related governmental policies.

Quantifying the importance of these factors is challenging because of the lack of data linkages at the individual level and the lack of variation in the timing and geographic coverage of many governmental policies. A limitation of this study is it analyzed only debts reported to TransUnion. It did not include debts not reported to credit bureaus, and our data may not be identical to debts reported to other credit bureaus.

## Notes

Supplement.
eMethods.
