# Supplementary material for: Trends in Medical Debt During the COVID-19 Pandemic
Source: JAMA Health Forum. 2022 May 20;3(5):e221031. doi: 10.1001/jamahealthforum.2022.1031 (PMC9123502; doi:10.1001/jamahealthforum.2022.1031)
Supplement: Supplement. — eMethods. [file jamahealthforum-e221031-s001.pdf]

Supplementary Online Content

Guttman-Kenney B, Kluender R, Mahoney N, Wong F, Xia X, Yin W. Trends in medical debt during the COVID-19 pandemic. *JAMA Health Forum.* 2022;3(5):e221031. doi:10.1001/jamahealthforum.2022.1031

eMethods.

This supplementary material has been provided by the authors to give readers additional information about their work.

© 2022 Guttman-Kenney B et al. *JAMA Health Forum.*

eMethods

We apply the sample restrictions and construct variables in the same manner as Kluender et al. (2021) when possible. Below we provide a summary of these sample and variable constructions decisions, along with full detail on variables new to this analysis.

1.1 TransUnion Credit Report Data

We use credit data provided by TransUnion, a global information solutions company, through a relationship with the Kilts Center for Marketing at The University of Chicago Booth School of Business.[1] The data are an anonymized, nationally representative 10% random sample of persons with credit reports.

1.2 Sample Restrictions

Other than updated timeframe, we apply the exact same sample restrictions as Kluender et al. (2021). We drop observations from prior to January 2018. We also drop persons who have a missing age or ZIP code, persons residing outside of the 50 states or the District of Columbia, and persons with an empty credit report, defined as a report with no credit record of any kind (i.e., no active or non-active tradeline, collection, or public record).

1.3 Variable Definitions

Medical and Non-Medical Debt in Collections: As in Kluender et al. (2021), we exclude medical debts that were in dispute, had been paid, or had been closed. Specifically, we define a medical debt in collections as a collection where i) Compliance Remark Code is not “AID”[2], ii) Current Balance Amount is positive, iii) Close Date is empty, and iv) the collection is classified as medical debt.[3] A nonmedical debt has the same definition as medical debt, except that it is classified as nonmedical.

We identify a collection by its Subject Key (the unique identifier for each person) and Collection Key. In the collection segment of TransUnion data, lines that have the same Subject Key and Collection Key in the same month are treated as duplicated collections, and only the line with the maximum Current Balance Amount among them is used.

Mean Flow of Medical and Non-Medical Debt in Collections: We first define the monthly flow of medical debt as the sum of new medical debt in a month. A new medical debt in collections is defined as a medical debt in collections that was not reported to TransUnion in the previous month. We inflation-adjust to September 2021 using the CPI-U. For comparability to Kluender et al. (2020), we Winsorize the person-month-level flow medical debt at the same 99.99^{th} percentiles from Kluender et al. (2020) of $184,590.98, which was calculated by pooling together new medical debt from all years from 2009 to 2020. We calculate the flow of non-medical debt in analogous manner, Winsorizing at $146,871.72. We calculate the mean monthly flows by dividing the flow by the total number of observations. To get a measure of the quarterly flow of medical debt for the analysis, we then aggregate the mean monthly flows to the quarterly level.

COVID-19 Infection Rate: We used the rolling 7-day average per 100,000 people from the New York Times.[4]

1.4 Pandemic severity measures

We examine the correlation between the percentage changes in medical debt and four measures of pandemic severity:

[1] TransUnion (the data provider) has the right to review the research before dissemination to ensure it accurately describes TransUnion data, does not disclose confidential information, and does not contain material it deems to be misleading or false regarding TransUnion, TransUnion’s partners, affiliates or customer base, or the consumer lending industry.
[2] In Compliance Remark Code, "AID" stands for "Account information disputed by consumer."
[3] This classifier variable takes on a value of 1 if the debt is “nonmedical” and 2 if the debt is “medical.”
[4] https://github.com/nytimes/covid-19-data.

© 2022 Guttman-Kenney B et al. *JAMA Health Forum.*

- The cumulative COVID-19 infection rate through September 1, 2021. We use the daily cumulative number of cases reported in each county across the U.S. divided by the population of that county. The infection data is from the New York Times,[5] and the population data is for 2019 and comes from the US Census Bureau.[6]
- The cumulative COVID-19 vaccination rate through September 1, 2021. We use the daily COVID-19 cumulative vaccination rate data for each county across the U.S. except those in Texas and Hawaii from Centers for Disease Control and Prevention.[7] The vaccination data for Texas is separately collected from Texas Department of State Health Services,[8] and the vaccination data for Hawaii is separately collected from State of Hawaii – Department of Health.[9] The underlying population data is for 2019 and comes from the US Census Bureau.[10]
- Change in the unemployment rate between September 2019 and the peak rate during the pandemic (April 2020 for most counties). We use the county-level seasonally adjusted unemployment data from the US Bureau of Labor Statistics.[11]
- Change in consumer spending between January 2020 and September 1, 2021. We use the aggregated and anonymized consumption data on consumer credit and debit card spending from Opportunity Insights.[12] The data is seasonally adjusted so that the measured change in consumption represents the change net of the average seasonal trends.

[5] https://github.com/nytimes/covid-19-data.
[6] https://www.census.gov/data/tables/time-series/demo/popest/2010s-counties-detail.html.
[7] https://data.cdc.gov/Vaccinations/COVID-19-Vaccinations-in-the-United-States-County/8xkx-amqh.
[8] https://tabexternal.dshs.texas.gov/t/THD/views/COVID-19VaccineinTexasDashboard/Summary?%3Aembed=y.
[9] https://health.hawaii.gov/coronavirusdisease2019/why-track-these-numbers/.
[10] https://www.census.gov/data/tables/time-series/demo/popest/2010s-counties-detail.html.
[11] https://download.bls.gov/pub/time.series/la/la.data.64.County.
[12] https://github.com/OpportunityInsights/EconomicTracker/blob/main/docs/oi_tracker_data_documentation.md.

© 2022 Guttman-Kenney B et al. *JAMA Health Forum.*

eReference

1. Kluender R, Mahoney N, Wong F, Yin W. Medical debt in the US, 2009-2020. *JAMA.* 2021;326(3):250–256.

© 2022 Guttman-Kenney B et al. *JAMA Health Forum.*
